# New and little known Isotomidae (Collembola) from the shore of Lake Baikal and saline lakes of continental Asia

**DOI:** 10.3897/zookeys.935.49363

**Published:** 2020-05-21

**Authors:** Mikhail Potapov, Cheng-Wang Huang, Ayuna Gulgenova, Yun-Xia Luan

**Affiliations:** 1 Senckenberg Museum of Natural History Görlitz, Am Museum 1, 02826 Görlitz, Germany Senckenberg Museum of Natural History Görlitz Görlitz Germany; 2 Moscow Pedagogical State University, Moscow, 129164, Kibalchicha St. 6 b. 5, Russia Moscow Pedagogical State University Moscow Russia; 3 Key Laboratory of Insect Developmental and Evolutionary Biology, CAS Center for Excellence in Molecular Plant Sciences, Chinese Academy of Sciences, Shanghai, 200032, China Center for Excellence in Molecular Plant Sciences, Chinese Academy of Sciences Shanghai China; 4 Banzarov Buryat State University, Ulan-Ude, 670000, Smolina St. 24a, Russia Banzarov Buryat State University Ulan-Ude Russia; 5 Guangdong Provincial Key Laboratory of Insect Developmental Biology and Applied Technology, Institute of Insect Science and Technology, School of Life Sciences, South China Normal University, Guangzhou, 510631, China South China Normal University Guangzhou China

**Keywords:** arid zone, endemism, fauna, sexual dimorphism, shingly beach, springtails

## Abstract

Collembola of the family Isotomidae from the shores of Lake Baikal and from six saline lake catenas of the Buryat Republic (Russia) and Inner Mongolia Province (China) were studied. *Pseudanurophorus barathrum* Potapov & Gulgenova, **sp. nov.** and *Parisotoma baicalica* Potapov & Gulgenova, **sp. nov.** from Baikal and *Ephemerotoma buryatica* Potapov, Huang & Gulgenova, **sp. nov.** and *Folsomia mongolica* Huang & Potapov, **sp. nov.** from saline lakes are described here. A morphological description of epitokous males of *Scutisotoma acorrelata* Potapov, Babenko & Fjellberg, 2006 is given. A list of 23 species of the family Isotomidae found in the shores of studied lakes is provided based on literature sources and newly collected material.

## Introduction

The springtail fauna of lake shores in Asia is poorly known. Some data is available from lakes of western Siberia (Stebaeva 1981, 2006; Berezina 2006) where changes in species composition along forest-steppe lakesides were studied. Basing on those materials several specialised species have been discovered at saline lakes (Stebaeva 1978). In 2014 and 2015 a Chinese-Russian team of researchers investigated the Collembola of more eastern areas of arid zone of continental Asia. Saline lands associated with six saline lakes of Buryatia (Russia) and Inner Mongolia (China) were studied resulting in the discovery of two new species.

Approximately 10–20 million years old Lake Baikal, a huge ancient reservoir of fresh water at the centrum of Asia, has exceptionally high faunal diversity and endemism (Martens 1997). Several new species of Collembola have been described from the lake shore (Potapov 1991; Babenko et al. 1994, 2011; Potapov et al. 2006; Huang and Potapov 2012; Potapov and Gulgenova 2013). Recent collecting on shingly beaches revealed two new species of the specialised littoral fauna of the lake.

Earlier, Sokolovskaya (1989) investigated the collembolan communities in the lower and sandy part of delta of Selenga River flowing into Baikal. Five species of Isotomidae were identified from several open and forest sites. Two were widely distributed species, *Isotoma viridis* Bourlet, 1839 and *Desoria olivacea* (Tullberg, 1871) and are not confirmed by us; *Proisotoma buddenbrocki* (Strenzke, 1954) (*Strenzketoma* Potapov, Babenko & Fjellberg, 2006 now) probably refers to *Scutisotoma acorrelata* Potapov, Babenko & Fjellberg, 2006 (see Table of Appendix 1).

In the present paper we describe four new species, two from saline lakes and two from Baikal, and provide a list of springtails of the family Isotomidae recorded from the surveyed lakes.

## Materials and methods

Collembola were sampled in catenas of six saline lakes in 2014 and 2015. Shores of the following lakes were studied: Alginskoye (53.633°N, 109.936°E), Nukhe-Nuur (54.027°N, 110.277°E) (Barguzin Valley, N Buryatia: Russia), Verkhneye Beloye (50.634°N, 105.720°E), Selenginskoye (51.356°N, 106.558°E) (Selenga Valley, SW Buryatia: Russia), Bayin Chagan Nuori (48.38°N, 118.71°E), and Hujiri Nuo Ergacha (48.30°N, 118.56°E) (E Inner Mongolia Province: China) (Fig. 58). Tullgren/Berlese funnels were used to extract Collembola from 492 soil cores, 125 cm^3^ each. Samples were collected from four positions at each catena: lowest accumulative part, two transit parts and upper alluvial part (steppe). One of the views of the catena at Lake Verkhneye Beloye is shown in Fig. 59. All parts were saline and covered with halophytes. On Lake Baikal shore, the springtails were collected in 2008–2017 by floatation in water of shingle and sand.

### Abbreviations

**A, B, C, D, E** papillae of labial palp;

**Abd.** abdominal segments;

**alt** altitude;

**Ant** antennal segments;

**AO** antennal organ;

**bms** basal ms on antennal segments;

**BSU**Banzarov Buryat State University;

**e-guards** supplementary setae for E-papilla of labium;

**G, H** ocelli G and H;

**ms** micro s-seta(e) (= microsensillum(a) auct.);

**MSPU**Moscow State Pedagogical University;

**p-row of setae** setae of posterior row;

**PAO** postantennal organ;

**s** in the text and figures macro **s**-seta or **s**-setae (= macrosensillum(a) or sensillum(a) auct.);

**SEM** Shanghai Entomological Museum;

**SMNG** Senckenberg Museum of Natural History Görlitz;

**Th** thoracic segments;

**Ti** tibiotarsi;

**U3** inner edge of unguis.

Types of new species are deposited in Moscow State Pedagogical University (Russia), Senckenberg Museum of Natural History Görlitz (Germany), Shanghai Entomological Museum (China), Banzarov Buryat State University (Russia). Cavity slides with Gisin’s liquid and flat slides with Hoyer’s medium were used to mount the specimens.

Notation of elements of labial palp follow Fjellberg (1999), elements of maxillary head follow Fjellberg (1984), labrum follow Yosii (1976), and chaetotaxy of p-row of tergites in *Parisotoma* Bagnall, 1940 follow Potapov (1991).

## Description of species

### Pseudanurophorus barathrum

Taxon classificationAnimaliaCollembolaIsotomidae

Potapov & Gulgenova sp. nov.

4AF91ACA-3655-5987-BAA8-39FB791F4D4C

http://zoobank.org/D3D18099-2978-4569-8E40-29419DA14E20

Figures 1–5 , 6–11 , 12–13

#### Type material.

***Holotype***: female. Buryat Republic, Severo-Baykalskiy District, ~ 30 km N Severobaykalsk, near Slyudanskoye Lake, 55.4627°N, 109.1698°E, shingly beach of Baikal, 17.VIII.2013, coll. M. Potapov and A. Gulgenova. 5 paratypes from the same place (Holotype and 2 paratypes deposited in MSPU, 3 – in SMNG). 2 paratypes from Russia, East Siberia. Irkutskaya Region, Slyudyanskiy District, Slyudyanka, shore of Lake Baikal, 51.6529°N, 103.7350°E, on moistened big stones (by aspirator), 29.VIII.2008, coll. M. Potapov (deposited in BSU).

#### Other material.

Irkutskaya Region, Slyudyanskiy District, Angasolskaya, shore of Lake Baikal, 51.7314°N, 103.8280°E, in shingle, 09.VIII.2015, coll. G. Efanov; Irkutskaya Region, Irkutskiy District, shore of Baikal, Primorskiy Range, Pribaikal’skiy Nat. Park, Khargino, 52.320°N, 105.776°E, stony beach, near water edge (by aspirator), 17.VIII.2013, coll. A. Babenko; Irkutskaya Region, Olkhonskiy District, Primorskiy Range, Kuyada, mouth of Talovka River, 52.553°N, 106.136°E, stony beach, under stones, 15.VII.2013, coll. A. Babenko; Buryat Republic, Kabansky District, Posolskiy Sor Bay, near Baykalskiy Priboy, 51.91216°N, 106.13954°E, seaweed debris near water, 23.VII.2011, coll. A. Chimitova and L. Vanyavina; Buryat Republic, Barguzinskiy District, 53.29645°N, 108.6213°E, floatation of shingle at water edge, 03.VIII.2014, coll. M. Potapov and A. Gulgenova. The materials are deposited in MSPU and SEM.

#### Description.

**Figures 1–5.**
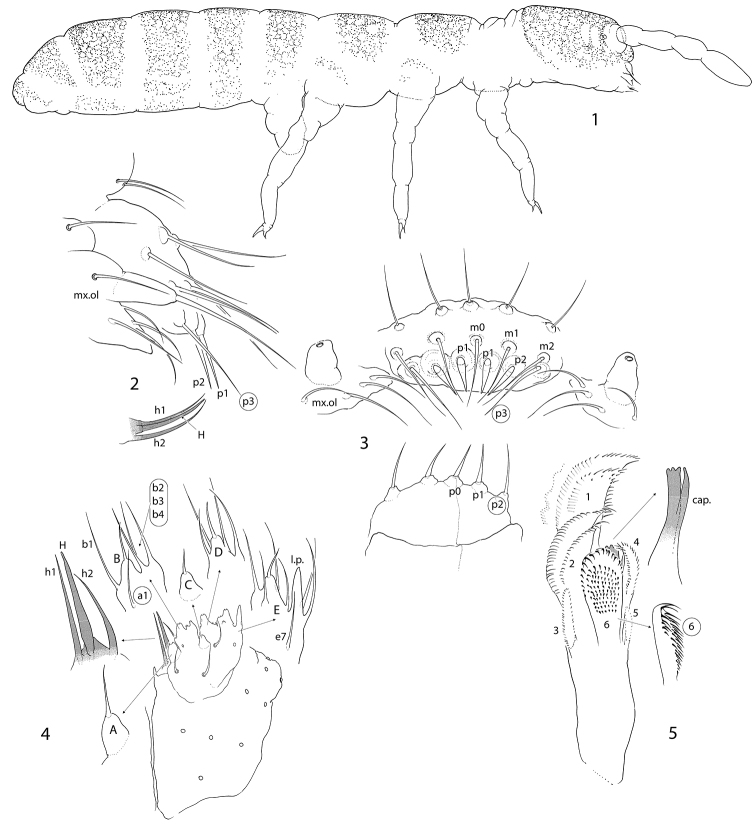
*Pseudanurophorus barathrum* sp. nov. **1** appearance of dark coloured specimen **2** labrum, maxillary outer lobe and hypostomal setae, lateral view **3** labrum and maxillary outer lobe, fronto-ventral and ventral views **4** labium, ventral view **5** maxillary head. Abbreviations: A, B, C, D, E-papillae of labial palp, H, h1, h2–hypostomal setae, mo, m1, m2, p1, p2, p3–setae of m – and p-row of labrum, mx.ol-maxillary outer lobe, cap., 1–6–capitulum and lamellae 1–6 of maxillary head, a1, b1, b2, b3, b4, e7–labial guards, l.p.–lateral processes. Characters of greater value encircled.

**Figures 6–11.**
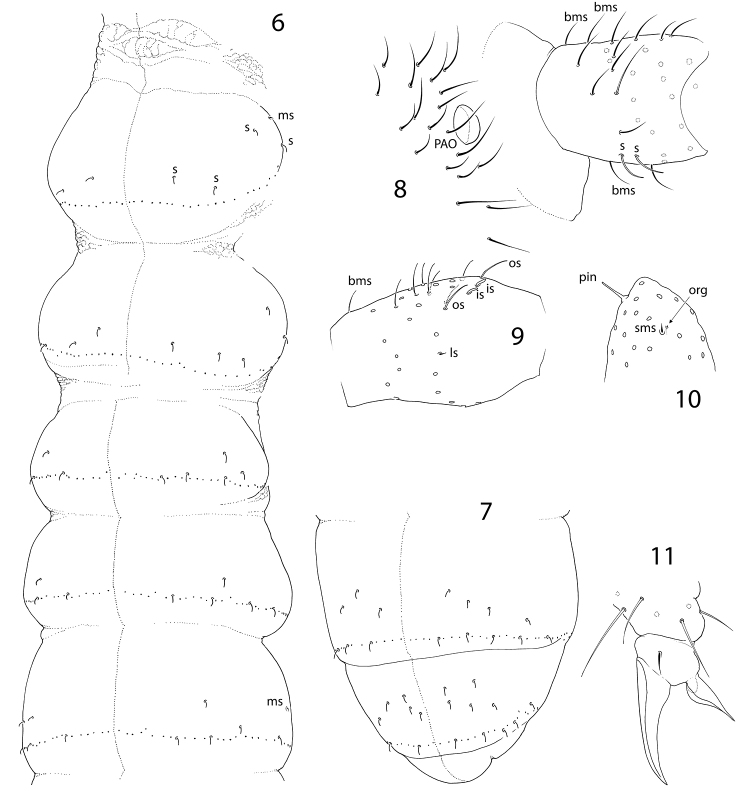
*Pseudanurophorus barathrum* sp. nov. **6, 7** s-setae on body in one variant **8**PAO and Ant.1, lateral view **9**Ant.3 (only sockets shown for setae, additional s-setae on inner side of segment not shown), lateral view **10** apex of Ant.4 (pin-seta, organite, and subapical ms shown) **11** apical part of Leg 2. Abbreviations: bms-basal micro s-seta, sms-subapical micro s-seta, org-organite, pin-pin-seta, s-s-seta, ms-micro s-seta, PAO-postantennal organ, is, ls, os-inner, lateral, and outer s-setae of antennal organ.

Size 1.0–1.5 mm. Body broad, with long legs (Fig. 1). Pigmentation from almost white to pale grey, forming a diffuse net interrupted by intersegmental areas. Cuticle finely reticulated (“smooth”), size of polygons much smaller than bases of setae. Large specimens sometimes with regularly scattered hardly visible small pits. Lateral parts of intersegmental area with secondary granulation (Fig. 6). Ocelli absent. PAO small, not constricted, ca. 0.3 as long as Ant.1 width and ca. half as long as U3 (Fig. 8). Maxillary outer lobe with simple maxillary palp and four slender sublobal hairs (Figs 2, 3). Labral formula as 3/556 (Fig. 3). Labral edge reduced, apical ridges absent. Apical row (p-row) of labral setae projecting above mouth aperture (Figs 2 and 3), normally with six setae: p1, p2, and p3 on each side, lateral pair (p3) more slender and long. Medial pair p1+p1 is sometimes replaced with one (p0) giving five setae (p0, p1, p2) in apical row and labral formula 3/555 (Fig. 3). Setae of two rows (m, p) of labrum and sublobal hairs form a basket surrounding the mouth. Labium with all papillae (A–E), papillae A without guards (Fig. 4). Guard a1 detached and integrated to papilla B which, in result, supplied with five guards (a1, b1–4). Guards b2, b3, b4 set together, on lateral expansion of papilla B. Papilla C without guards, D with four guards in normal position. E with seven guards, lateral process and two lateral guards enlarged. Terminal setae of all papilla short. All elements of hypostomal group (H, h1, h2) considerably enlarged and bent towards labrum complementing the upper “basket” (Figs 2, 4). Main part of labium with three proximal, four basomedian and five basolateral setae. Maxillary head modified: capitulum slender and formed by fused claws, lamellae enlarged. Lamellae 1 and 2 well beyond capitulum, each supplied with apical and inner rows of cilia. Outer edge of lamella 1 gently ciliated, sometimes wavy. Lamella 3 expanded, with strong teeth (Fig. 5). Ventral side of head with 5-6+5-6 postlabial setae. S-setae and bms-setae of antennae slender and resemble common setae. Ant.1 with two basal, ventral and dorsal, bms, two ventro-lateral s, and ca. 30–40 common setae (Fig. 8). Ant.2 with three bms and one laterodistal s. Ant.3 with one bms, five common s (two outer, two inner, and one lateral spine-like), and a group of additional thin s-setae located on dorsal and inner sides of segment (Fig. 9), inner additional s-setae hardly differ from common setae. S-setae on Ant.4 weakly differentiated, subapical organite rudimental, apex without bulb (Fig. 10).

Body with numerous smooth short setae. Dorsal axial setal pattern asymmetric, can approximately be described as: 12–14,11–14/9–10,9–10,9–10,12–14 (Th.II–Abd.IV). Macrosetae not differentiated. S-setae on tergites weakly differentiated, subequal to common setae (Fig. 13). S-setae varies as 4,4–5/3–5,4–5,4–6, ~9, ~10, number and arrangement asymmetrical. With incomplete set of ms-setae (1,0/0,0,1). Most s-setae on Abd.I–Abd.V in p-row of setae (Figs 6, 7, 13). Th.I, II, and III without ventral setae.

**Figures 12, 13.**
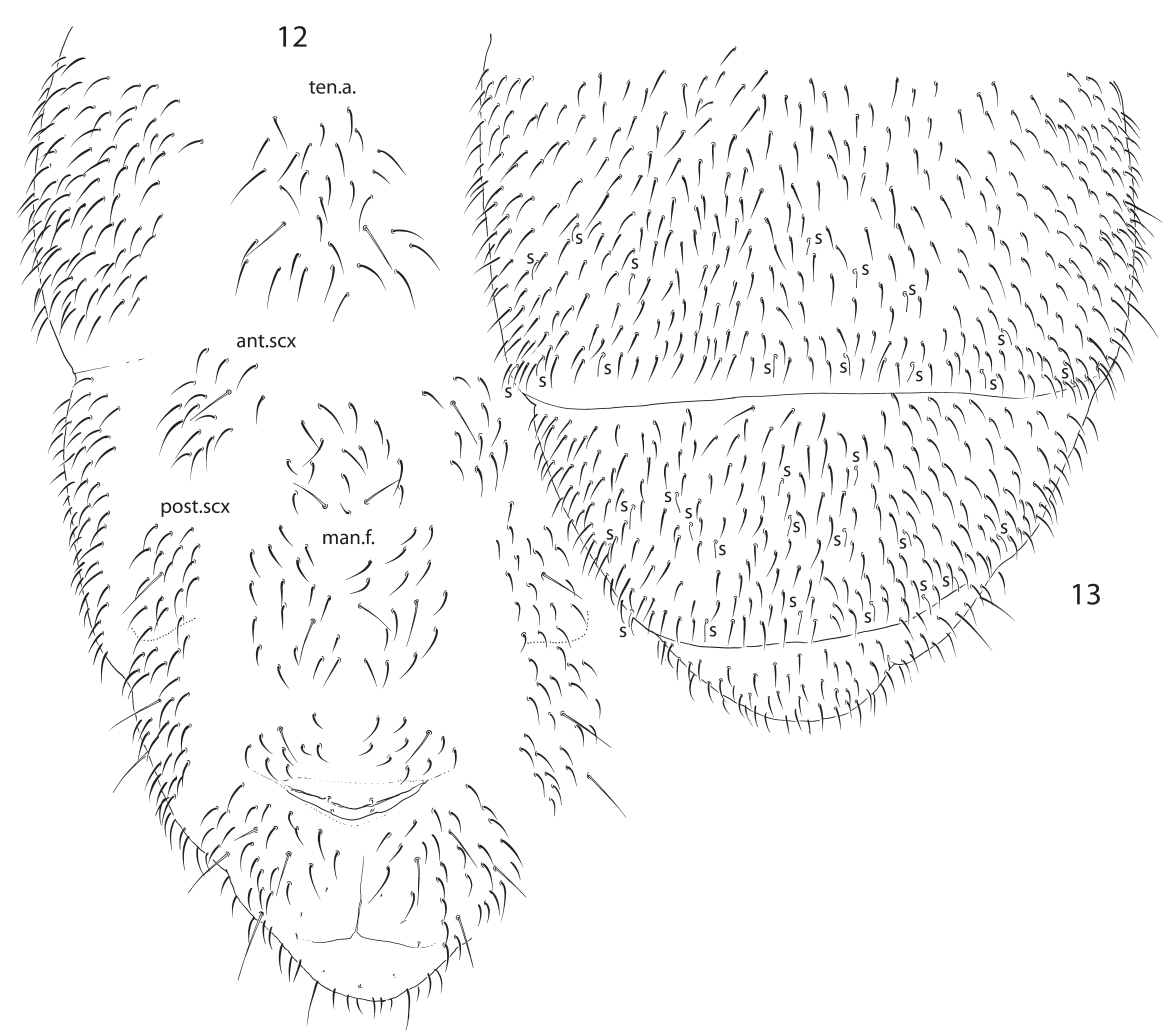
*Pseudanurophorus barathrum* sp. nov., chaetotaxy of ventrum of Abd.III–VI (**12**) and dorsum of Abd.IV–VI (**13**) Abbreviations: ten.a.–tenacular area, man.f.–manubrial field, ant.scx, post.scx-anterior and posterior subcoxae, s-s-seta.

Unguis rather slender, without teeth (Fig. 11). Empodial appendage with lamellae, without teeth. All tibiotarsi with many additional setae, ca. 40 on Ti.1 and Ti.2 and 50–60 on Ti.3. Adult male often with slightly swollen Ti.3, with stick-like thin spurs (x and B5). Tibiotarsi of all legs with seven setae in apical whorl. Tibiotarsal tenent setae pointed (Fig. 11). Ventral tube with 12–14+12–14 distal and ca. 20 posterior setae, without anterior setae. Distal setae in two groups, anterior (9–11+9–11) and posterior (3+3). Tenaculum and furca entirely absent. Tenacular field with ca. 20–30, manubrial field with ca. 40–50 setae (Fig. 12). Manubrial setae in anterior and posterior groups. Anterior furcal subcoxae with 13–16 setae, posterior subcoxae with 18–20 setae. Posterior subcoxa weakly separated from setaceous part of segment. Males present.

#### Affinity.

This remarkable species is characterised by two (more rarely one) additional setae on labrum that is so far unknown in *Pseudanurophorus* Stach, 1922 and other Collembola as well. Labral formula 5,5,4 (vs. 5,5,5–6 in *P. barathrum* sp. nov.) is invariable in Isotomidae and it is clear that the character is often omitted although being implied in the descriptions. Other mouth parts are also strongly modified: labral edge is reduced; two anterior setal rows and the sublobal hairs form a basket surrounding the mouth; hypostomal setae and lateral process are enlarged; guard a1 is integrated with papilla B; guards b2, b3, b4 set on lateral expansion of B; maxillary head has slender capitulum and expanded lamella. Unusually high number of setae on tibiotarsi and ventral tube and the dense and short abdominal hair cover is an apparent adaptation to live in close contact with water, as in many other littoral species.

The new species belongs to the “*boerneri*” group due to three prelabral, 4+4 or more postlabial setae, simple maxillary palp and other characters (for details see Potapov 1997). It most resembles two species with short macrosetae *P. arcticus* Christiansen, 1952 and *P. montanus* Martynova, 1971 (1971a), for which labral formula is unknown. Following the first description and Fjellberg’s comments (1975) on *P. arcticus* paratypes, these two species have much fewer setae in axial group of tergites and yet have macrosetae on last abdominal segments (vs. absent in *P. barathrum* sp. nov.). *Pseudanurophorus arcticus* is described and subsequently recorded in the Arctic and *P. montanus* in the mountains of Middle Asia.

#### Distribution and ecology.

Several records from the littoral zone of the shore of Lake Baikal, none found inland. It is one of the common species in shingly beaches.

#### Name derivation.

It is named after the specific mouth parts (*barathrum* – a glutton in Latin, among other translations).

### Scutisotoma acorrelata

Taxon classificationAnimaliaCollembolaIsotomidae

Potapov, Babenko & Fjellberg, 2006

95A19AC4-A00D-5EC8-A9F9-52CB03F33FA4

Figs 14–17

#### Material.

37 specimens. Russia, Buryat Republic, Eravninsky District, coast of Bolshaya Eravna Lake, 06.VI.2008, coll. A. Gulgenova; Buryat Republic, Kabansky District, shore of Lake Baikal, Posolskiy Sor Bay, near Baykalskiy Priboy, 51.91216°N, 106.13954°E, seaweed debris near water, 23.VIII.2011, coll. A. Chimitova and L. Vanyavina; Buryat Republic, Barguzin Valley, Alginskoye Lake, floatation of wet coarse sand and shingle at water edge, 03.VIII.2014, coll. M. Potapov, C.W. Huang, and A. Gulgenova.

#### Description of epitokous males with fully developed ejaculatory duct in shore of Lake Baikal.

**Figures 14–18.**
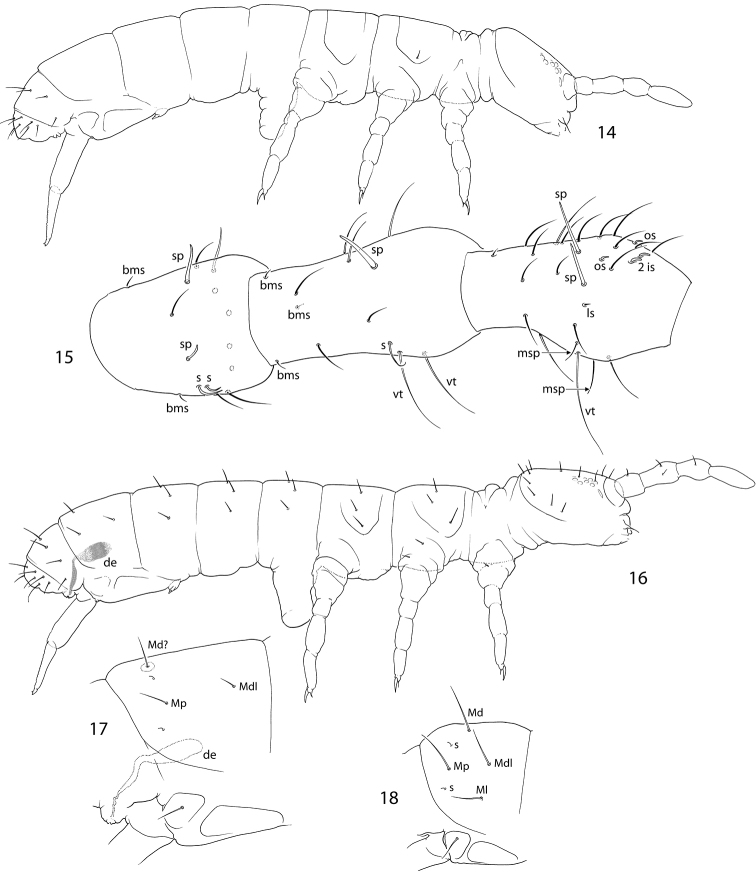
*Scutisotoma acorrelata***14, 16** appearance of adult female (14) and adult male in reproductive stage (16) (pigmentation not shown) **15**Ant.1–3 in adult male in reproductive stage, lateral view **17** macrosetae on Abd.IV in adult male of *S. acorrelata***18** common arrangement of macrosetae on Abd.IV in Anurophorinae. Abbreviations: bms-basal micro s-seta, de-ductus ejaculatorius, s-s-seta, is, ls, os-inner, lateral, and outer s-setae of antennal organ, sp-spiny setae, vt-ventral trichobothrium, msp-male spurs, Md, Mdl, Mp, Ml-macrosetae of Abd.IV.

Size ca. 1.2 mm, subequal to adult females. Macrosetae erect, slightly serrated, well developed on all body tergites and head. Three first segments of antennae with thickened setae (Fig. 16). In females, subadult males and juveniles macrosetae are only developed on thoracic (only lateral pair) and two last abdominal segments (Fig. 14). Number of macrosetae 3,3/3,3,3,3, their arrangement as common for Anurophorinae (in position Md, Mdl and Ml), apart from Abd.IV on which Mp and Mdl are in common position while macrosetae Ml is absent and Md shifted backwards and set in posterior row of setae. Common arrangement of macrosetae on Abd.IV for Anurophorinae shown in Fig. 18. Ventral side of Abd.VI with two thin curved macrosetae (vs. of normal shape in females). Head with macrosetae at posterior edge, in ocellar field, and between antennae. Front of head slightly swollen (vary). Antennae bent downwards. Three first segments of antennae thickened. In fully developed variant Ant.1 with three spiny setae (sp), Ant.2 with one sp and two ventral trichobothria, Ant.3 with two sp, one ventral trichobothrium and few (two or three) male “spurs” (Fig. 15). Thicknesses of sp vary. Tibiotarsus 3 with setae X and B5 insignificantly modified, set in wider sockets than in female.

#### Remarks.

Considering the dimorphic species *S. muriphila* (Grinbergs, 1968) and *S. stepposa* (Martynova, 1975) (for details see Grinbergs (1968), Chimitova and Potapov (2011)), *S. acorrelata* is the third member for the genus *Scutisotoma* Bagnall, 1948 which shows well developed sexual dimorphism. Males of *S. acorrelata* are less modified than in the two other species and polymorphism was not evident in our material. In taxonomical terms, the presence of epitokous males is a character of low value at generic level. It is probably optional in several genera. At species level, the diagnostic value of epitokous males is questionable. In many genera of the family Isotomidae the epitokous males are probably more frequent than usually considered. The short duration of the reproductive instar may have left many epitokous forms undetected.

#### Distribution and ecology.

The species was described from shore of Lake Baikal (Potapov et al., 2006) and was further recorded at saline Alginskoye Lake and freshwater Bolshaya Eravna Lake. It lives in seaweed debris and in coarse sand.

### Ephemerotoma buryatica

Taxon classificationAnimaliaCollembolaIsotomidae

Potapov, Huang & Gulgenova sp. nov.

EF0EAB43-F494-5904-9F98-792600800CEA

http://zoobank.org/01C359C5-F519-4B0A-9B06-8445933FD6E0

Figures 19–26 , 27–31

#### Type material.

***Holotype***: female. Russia, south-western part of Buryat Republic, Gusinoozerskaya Basin, 0.5 km SW from Tokhoy, 51.356417°N, 106.558733°E, 590 m alt., southern shore of Sul’phatnoye (= Selenginskoye) Lake, grassland with *Caragana* sp., *Achnatherum splendens*, *Atriplex* sp., *Leymus* sp., 18.X.2015, coll. M. Potapov and A. Gulgenova. 24 paratypes (sub-adult and adult males and females) from the same place. Holotype and 10 paratypes deposited in MSPU, 4 in BSU, 5 in SMNG, 5 in SEM.

#### Other material.

From the type locality dated 02.V.2015 and 25.VII.2015.

#### Description.

Size 0.6–0.9 mm. Body as common for Anurophorinae with short furca (Fig. 27). Pigmentation grey, as in *Proisotoma minuta* (Tullberg, 1871). Cuticle finely reticulated, size of largest polygons smaller than bases of setae. Ocelli 8+8, G and H smaller (Fig. 23), all ocelli usually look subequal by pigmentation. PAO with three guard setae along posterior margin, elliptical, not constricted, as long as 0.4–0.6 of Ant.1 width and 0.7–1.1 as long as U3 (Fig. 23). Maxillary outer lobe with simple maxillary palp and four sublobal hairs. Labral formula as 2/554. Labium with all papillae (A–E), papillae A–D with normal number of guards (1,4,0,4), E with four guards (Fig. 21). Main part of labium with three proximal, four basomedian and five basolateral setae. Ventral side of head with 4+4 postlabial setae. Ant.1 with two basal, ventral and dorsal, bms, two ventro-lateral s, and eleven setae, without p-setae (Fig. 23). Ant.2 with three bms and one laterodistal s. Ant.3 without bms and with five distal s (including one lateral spine-like), inner s of AO small (Fig. 22). All s-setae on Ant.1–3 very short. S-setae on Ant.4 weakly differentiated, subapical organite small. Apex of Ant.4 with bilobed bulb (Figs 24–26), well visible in dorsal view (Fig. 25). In fully grown animals the bulb can look trilobed due to slight secondary division of one of the lobes (Fig. 26).

**Figures 19–26.**
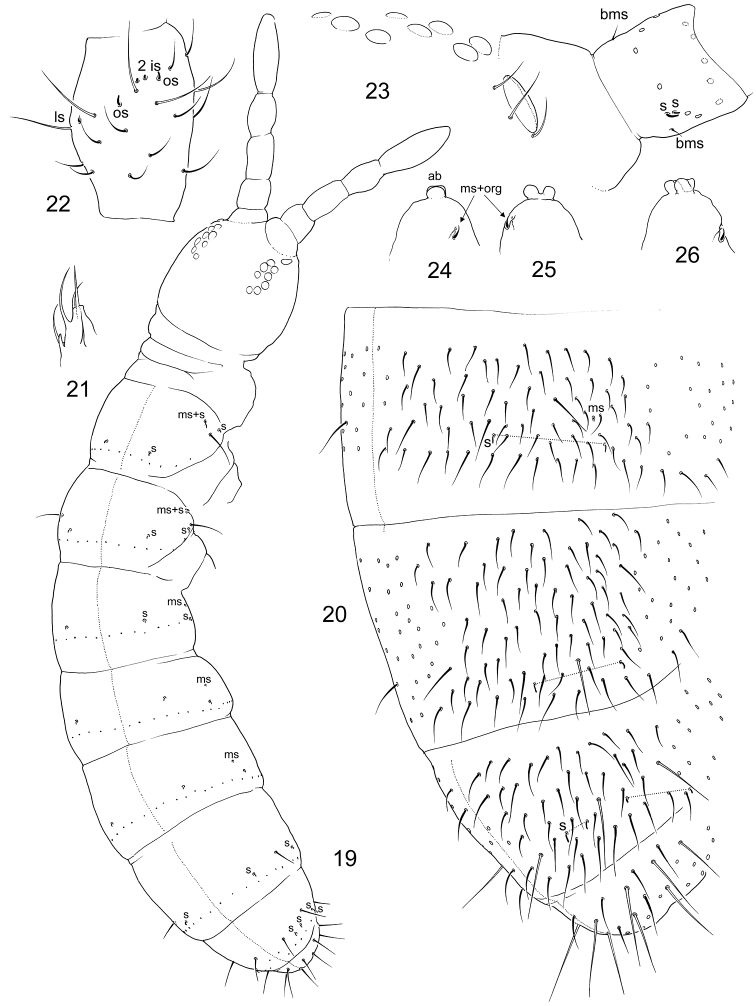
*Ephemerotoma buryatica* sp. nov. **19** s-, ms-setae, macrosetae, and p-rows of setae on body tergites **20** chaetotaxy of Abd.III–VI **21** labial papilla E **22**Ant.3, dorso-lateral view **23** ommatidia and Ant.1 **24–26** apex of Ant.4, lateral (**24**), dorsal (**25**), and ventro-lateral (**26**) views. Abbreviations: bms-basal micro s-seta, ab-apical bulb, ms-micro s-seta, org-organite, s-s-seta, is, ls, os-inner, lateral, and outer s-setae of antennal organ.

Body with smooth and rather short setae. Dorsal axial setal pattern of Th.II–Abd.IV: 7–8,6–7/5,5,5,7–8. Th.I and II without ventral setae, Th.III with 3–5+3–5 (usually 4+4) ventral axial setae (Fig. 31). Abd.II with a pair of mid-ventral setae (Fig. 31). Macrosetae weakly differentiated, medial macrosetae on Abd.V as long as 0.25–0.35 of tergal midline (Fig. 20). S-setae on tergites very short (Fig. 20). S-formula as 3,3/2,2,2,2,4 (s) and 1,1/1,1,1 (ms). In al-group of Th.II and III front s-seta and ms-seta set close to each other (notated as ‘ms+s’ in Fig. 21). S-setae on Th.II–Abd.V set in front of p-row of setae (Fig. 1). On Abd.V s-setae arranged in one transverse row, lateral pair very short (Fig. 2).

**Figures 27–31.**
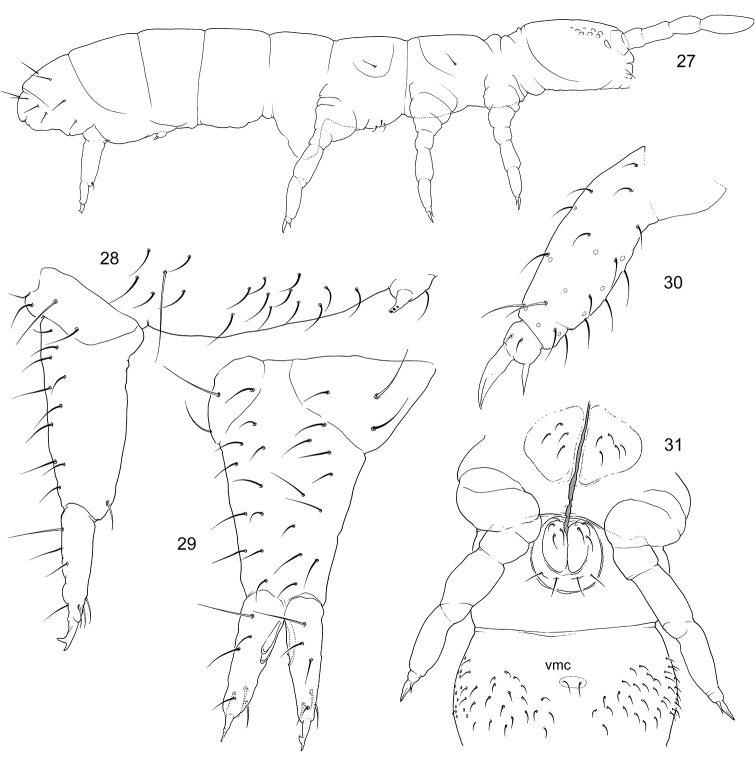
*Ephemerotoma buryatica* sp. nov. **27** appearance **28** furcal area, lateral view **29** furca, posterior view **30** tibiotarsus of Leg 3, outer view **31** ventral side of Th.II and Abd.I and II. Abbreviations: vmc-ventro-medial setae.

Unguis of unusual shape, expanded at the middle, without teeth. Empodial appendage slender, without lamellae. Ti.1 and Ti.2 without additional setae (21), Ti.3 usually with 25 setae. B-row of setae on Ti.1–2 complete (B4 and B5 present). Male spurs (x and B5 on Ti.3) in adult males thin, stick-like. Tibiotarsal tenent setae pointed (Fig. 30). Ventral tube with 4+4 distal and four posterior setae arranged in one transversal row (Fig. 31). Tenaculum with 3+3 teeth and one seta. Anterior furcal subcoxae with 10–13 setae, posterior subcoxae with five or six setae (Fig. 28). Anterior side of manubrium with a pair of distal setae (Fig. 28), posterior side with 9–10+9–10 setae on main part, lateral edges without setae (Fig. 29), laterobasal lobes with 3+3 setae. Dens short, about half as along as Ti.3. Anterior side of dens with three setae in subapical position (Figs 28, 29). Posterior side of dens usually with large subapical hump, more proximal part without clear modifications. Dens with four posterior setae, three in basal half, one subapical. Mucro with two teeth, subapical tooth largest. Ratio of manubrium : dens : mucro = 5.5–6.5 : 2.4–3.3 : 1.0. Males present.

#### Affinity.

The species belongs to recently described genus *Ephemerotoma* Potapov, Kahrarian, Deharveng & Shayanmehr, 2015 due to simple maxillary palp, reduced number (four) of guards on labial papilla E, two prelabral setae, complete set of ms-setae on tergites (11/111), and tergal s-setae on abdomen set in front of p-row. *Ephemerotoma buryatica* sp. nov. does not share a significant character of the genus, the “two transverse rows” pattern of s-setae on Abd.V. The sexual dimorphism common for the genus *Ephemerotoma* [*E. porcella* (Ellis, 1976), *E. skarzynskii* Potapov, Kahrarian, Deharveng & Shayanmehr, 2015, *E. huadongensis* (Chen, 1985)] is not observed in the new species. Small and rather slender body, short furca, shape of unguis, and absence of sexual dimorphism indicate a preference for deeper edaphic habitat than in its congeners.

Regarding all genera of the *Proisotoma* complex, a peculiarity of the new species is the bilobed apical bulb on Ant.4, which is otherwise known only in *Proisotoma bulba* Christiansen & Bellinger, 1980 (California, U.S.A.). The generic position of *P. bulba* is obscure because of lack of information on mouth parts and s-setae on body. In other characters, *E. buryatica* sp. nov. differs from *P. bulba* by fewer setae on dens (3/4 vs. 4–5/5–6), shorter dens (dens : mucro = 9 : 1 in *bulba*), teeth on tenaculum (3+3 vs. 4+4) and characters of unguis and tibiotarsi (*bulba* has inner tooth on unguis and a clavate tenent hair). In *Proisotoma* complex, a similar furca is shown, for example, for *Weberacantha echinodermata* Potapov, Babenko & Fjellberg, 2006 and *Scutisotoma robustodens* Huang & Potapov, 2012, which belong to other genera. Mouth parts (two prelabral setae, simple maxillary palp and reduced number of e-guards) of *E. buryatica* sp. nov. resemble the “*asiatica*” group of the genus *Subisotoma* but several other characters of great value are different (e.g., presence/absence of anterior setae on manubrium).

#### Distribution and ecology.

Known only from one locality in SW Buryatia where it inhabits soil of dry steppe at upper part of a salt-lake catena. The species probably occurs in all seasons since it was recorded in May, July and October in the type locality. It was highly aggregated in October which that suggests a resemblance to the “ephemeral” species of the genus *Ephemerotoma*.

#### Name derivation.

It is named after the type locality.

### Folsomia mongolica

Taxon classificationAnimaliaCollembolaIsotomidae

Huang & Potapov sp. nov.

9571C78E-C755-5982-84B6-DCE601677E37

http://zoobank.org/F2435043-7D30-4D2D-911C-18F53B719173

Figures 32–39 , 40–42

#### Type material.

***Holotype***: female. NE China, E Inner Mongolia Autonomous Region, Hulun Buir, New Barag Zuoqi, Xinbaoligexi Sumu, Bayin Chagan Nuori Lake, at shore of the saline lake, 48.38°N, 118.71°E, 669 m alt., 09.VIII.2014, coll. C.W. Huang and M. Potapov. 20 paratypes from the same place. Holotype and 10 paratypes deposited in SEM, 5 – in MSPU, 5 – in SMNG.

#### Other material.

NW China, E Inner Mongolia Autonomous Region, Hulun Buir, New Barag Zuoqi, Xinbaoligexi Sumu, Hujiri Nuo Ergacha Lake, at shore of the saline lake, 48.30°N, 118.56°E, 649 m alt., 09.VIII.2014, coll. C.W. Huang and M. Potapov. China, W Inner Mongolia Autonomous Region, Helan Mts., near Halawu, mixed sample from broadleaved bush and coniferous trees, 2325 m alt., 08.VIII.2010, coll. C.W. Huang and Y. Bu.

#### Description.

**Figures 32–39.**
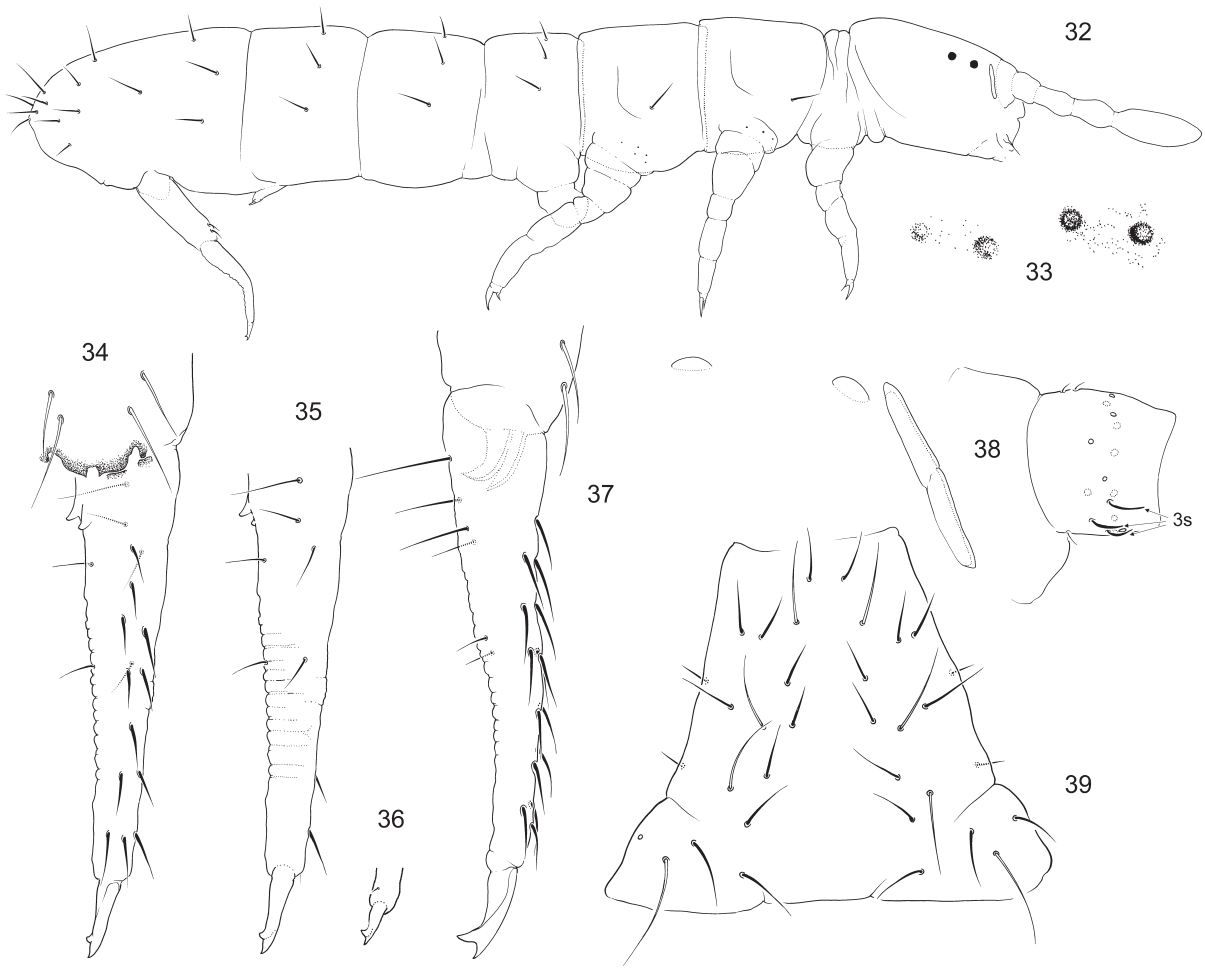
*Folsomia mongolica* sp. nov. **32** appearance **33** pigmention of eye areas (two different specimens) **34** dens and distal part of manubrium, anterior view **35, 37** dens, posterior (**35**) and lateral (**37**) views **36** distal part of dens and mucro, another specimen, posterior view **38** ommatidia, PAO, and Ant.1 **39** manubrium, posterior view. Abbreviations: s-s-seta.

Size 1.0–1.3 mm. Body of normal shape (Fig. 32). Usually without pigmentation apart from two contrasting black ommatidia on each side of head (Figs 32, 33). Darker specimens with diffuse black grains also on head and trunk. Specimens with weak eye pigmentation sometimes occur among normal ones, while cornea of ocelli are still distinct. Juveniles almost unpigmented. Cuticle with weak hexagonal primary granulation (“smooth”), thin belts of courser granulations at posterior edge of head, between Abd.IV and V and on medial line of thorax. Two widely separated large subequal ocelli on each side of head, like in *F. quadrioculata* (Tullberg, 1871) (Fig. 38). PAO narrow, well constricted, 1.1–1.4 as long as width of Ant.1 and 1.6–1.9 as long as inner unguis length. Maxillary outer lobe with four sublobal hairs, maxillary palp simple. Labral formula as 4/554. Labium with five papillae (А–Е) and full set of guard setae (e7 present), with three proximal and four basomedian setae. Ventral side of a head with 4–5+4–5 postlabial setae. Ant.1 with three ventral s-setae and three short basal ms-setae (bms), two dorsal and one ventral (Fig. 38), Ant.2 with three bms and one latero-distal s, Ant.3 with one bms and with five distal s (including one lateral), without additional s-setae. S-setae on Ant.4 weakly differentiated. Organite small.

**Figures 40–42.**
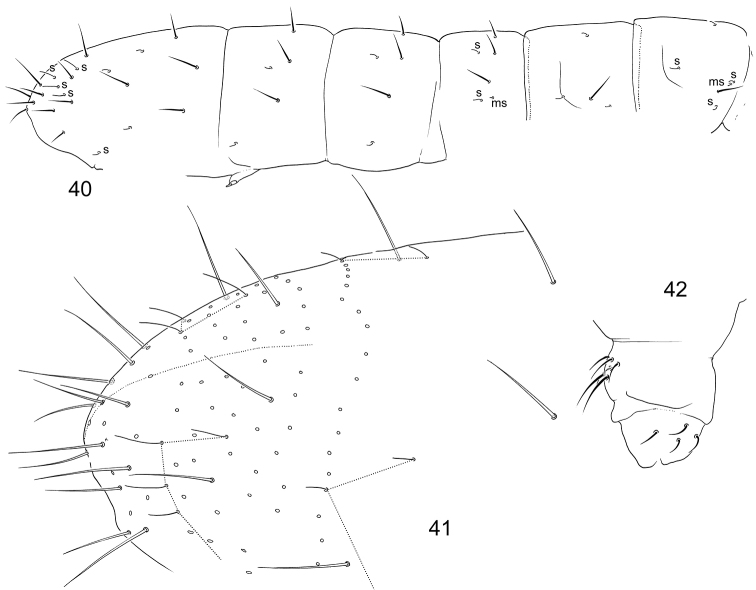
*Folsomia mongolica* sp. nov. **40** s-, ms-setae, and macrosetae on body tergites **41** s-setae and macrosetae of Abd.IV–VI (common setae shown as sockets, only p-row of setae presented on Abd.IV) **42** ventral tube, lateral view. Abbreviations: s-s-seta, ms-micro s-seta.

Macrosetae smooth and short, 1,1/3,3,3 in number (Fig. 40), medial ones on Abd.V 0.4–0.5 as long as dens and 2.0–3.1 as long as mucro. No foil setae at the tip of abdomen. Axial chaetotaxy as 8–10,7–8 /5,5,4–6 for Th.II–Abd.III. Thorax without ventral setae. S-formula as 4,3/2,2,2,3,5 (s), 1,0/1,0,0 (ms) (Fig. 40). Tergal s-setae as long and wide as common setae and hard to observe. Medial s-setae on Th.II–Abd.III situated in mid-tergal position, on Abd.I between Mac1 and Mac2, on Abd.II and III behind Mac2 (Fig. 40). Abd.V with five s-setae arranged as three dorsal ones (al, accp1, accp2), middle-sized and slender and one lateral (accp3) 2/3 as long as dorsal, and one ventral (the shortest, Fig. 41).

Unguis of normal shape, without lateral and inner teeth. Empodial appendage about half as long as unguis. Tibiotarsi with few additional setae on Legs 1 and 2 (23–25 setae), Leg 3 more polychaetotic. Tibiotarsal tenent setae pointed. VT with 4+4 (3+3 in small juveniles) laterodistal and six posterior setae, no anterior setae (Fig. 42). Tenaculum with 4+4 teeth and one seta. Anterior furcal subcoxae with 12–20 setae, posterior one with 4–6. Anterior side of manubrium with 2+2 setae (Figs 34, 37). Posterior side of manubrium with 4+4 laterobasal, two apical setae (ap), 3+3 setae in distal transversal row (M1, m1, L1), and 6–7+6–7 in central part (Fig. 39). Two pairs of lateral setae present. Dens with 12–14 (rarely 11 or 15) anterior setae (Figs 34, 37). Posterior side of dens crenulated, with seven setae: four setae at base one of which larger, two at middle part, and one rudimentary subapical seta which is often absent or hardly seen (Figs 35, 36). Mucro bidentate. Manubrium a little shorter than dens. Ratio of manubrium : dens : mucro = 3,9–5,4 : 4,7–6,2 : 1. Males present.

#### Affinity.

The species belongs to “*heterocellata*” group due to simple maxillary palp. *F. mongolica* sp. nov. is very similar to two other species inhabiting arid landscapes of continental Asia: *F. pseudodecemoculata* Stebaeva, 1971 and *F. heterocellata* Stebaeva & Potapov, 1997. All three forms have no body pigmentation and share several important characters: structure of furca, body chaetotaxy, number of s-setae on antennae. The only sharp difference is number of ocelli on each side of the head: two in *F. mongolica* sp. nov., four in *F. heterocellata*, and five in *F. pseudodecemoculata*. The last species has shorter PAO than in the new species. *F. montana* Martynova, 1971 (1971b) (high mountains plateaus of Kirghisia) also belongs to “*heterocellata*” group and has 2+2 ocelli, but differs by three basal setae on posterior side of dens (vs. four in *F. mongolica* sp. nov.), 3+3 (vs. 4+4) laterobasal setae on posterior side of manubrium, and shorter PAO.

#### Distribution and ecology.

The species is probably distributed in Inner Mongolia (China). This halophilic species is abundant on saline lands but also inhabits dry forest slopes.

#### Name derivation.

It is named after the location of type place (Inner Mongolia Autonomous Region).

### Parisotoma baicalica

Taxon classificationAnimaliaCollembolaIsotomidae

Potapov & Gulgenova sp. nov.

98E20638-EF17-52CF-B92E-71ADD92DFBF9

http://zoobank.org/34334075-2F6C-48F6-BC8E-3D15FCAF5222

Figures 43 , 44–57

#### Type material.

***Holotype***: female. Russia, East Siberia, Irkutskaya Region, Slyudyanka District, Angasolskaya, shore of Lake Baikal, 51.7314°N, 103.8280°E, in shingle, 09.VIII.2015, coll. G. Efanov (deposited in MSPU). 3 paratypes from the same place (deposited in MSPU), 4 paratypes from Russia, Buryat Republic, Barguzinskiy District, 53.29645°N, 108.6213°E, floatation of shingle at water edge, 03.VIII.2014, coll. M. Potapov and A. Gulgenova (2 paratypes deposited in SMNG, 2 – in BSU).

#### Description.

**Figure 43.**
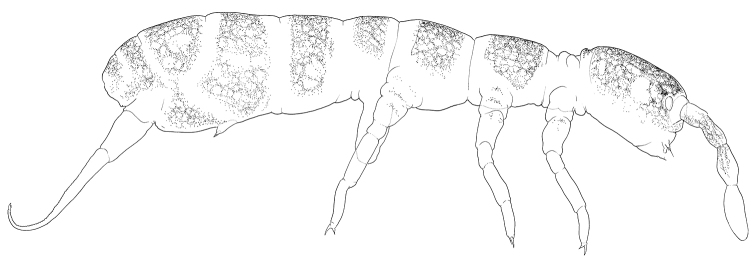
Appearance of *Parisotoma baicalica* sp. nov.

**Figures 44–57.**
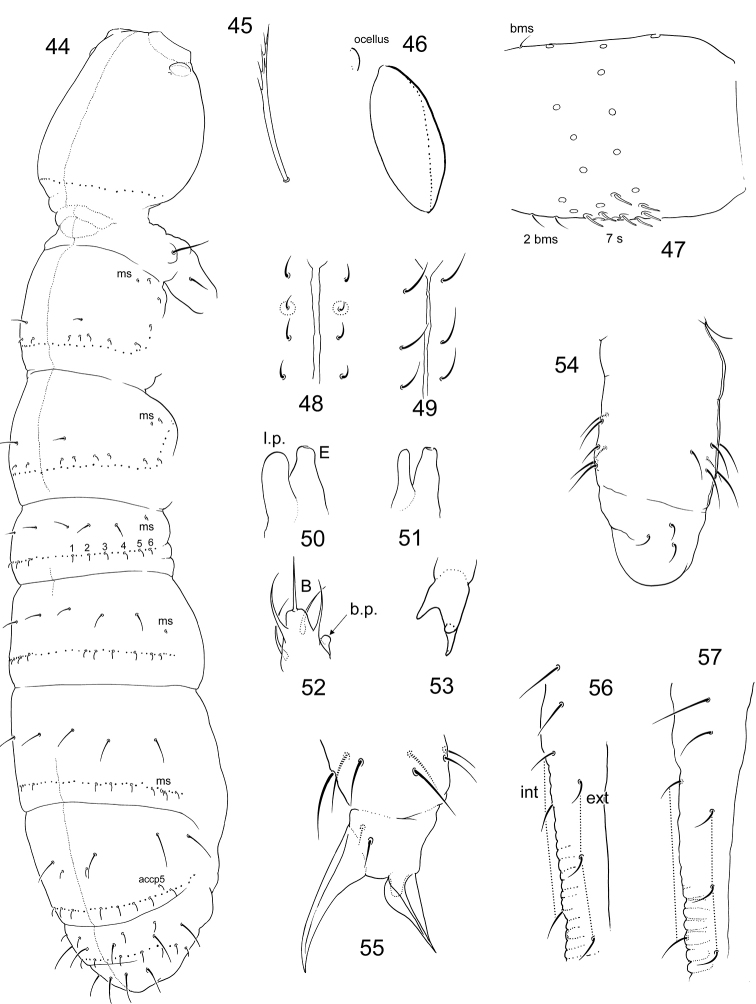
*Parisotoma baicalica* sp. nov. (**44–50**), (**52–55**) and *P. reducta* (**51, 56**) **44** s-, ms-setae, and macrosetae on body **45** macroseta of Abd.V **46**PAO and ocellus **47**Ant.1, lateral view (s and ms shown) **48, 49** postlabial setae, variation **50, 51** papilla E of labial palp, lateral view **52** papilla B, ventral view **53** mucro **54** ventral tube, lateral view **55** apical part of Leg 3 **56, 57** posterior side of dens. Abbreviations: s-s-seta, ms-micro s-seta, bms-basal micro s-seta, l.p.–lateral processes, b.p.–basal processes, B, E-papillae of labium, int, ext-internal and external setae of posterior side of dens.

Body length from 0,7 to 0,9 mm. Pale with diffuse greyish pigment on body, eye spot less marked than in most species of *Parisotoma* with one ocellus (Fig. 43). Ant.1 with 5–7 short s-setae ventro-laterally, three basal microsetae, two dorsal and one ventral (Fig. 47). Inner s-setae of AO III large. Ant.4 as common for the genus. One small ocellus on each side of head (Fig. 46). PAO wide, 1.4–1.8 as long as internal crest of Claw 3. Labral formula 4/554, apical folds sharp, as in *P. notabilis* (Schäffer, 1896). Maxillary outer lobe with four sublobal hairs and trifurcate apical palp. Labial palp with five papillae (A–E) and full set of guards (16, including e7), lateral process expanded. Papilla B with small basal process on its inner side (Fig. 52) (see the remarks). Labium with five basomedian, five basolateral, and four proximal setae. Number of postlabial setae from 3+3 to 4+4 (Figs 48 and 49), in the latter case an additional pair set between a1 and m1 (marked in Fig. 48). Inner mouthparts as usual for the genus: lamella 1 longer than capitulum with apex fan-shaped expanded, with marginal ciliation and one row of long denticles on inner side, lamella 6 with marginal ciliation and several (>3) irregular rows of denticles. Lower subcoxa of Leg 1 with one outer seta (Fig. 44). Tibiotarsi of all legs with only seven setae in apical whorl. Claw slender, without clear teeth (Fig. 55). Empodial appendage with broad lamella. Ventral tube with 3+3 lateral, 3+3 anterior (rarely two or four), and 4–6 posterior setae (Fig. 54). Retinaculum with 4+4 teeth and 2(3) setae. Furcal subcoxa with 27–35 setae. Manubrial thickening simple. Anterior side of manubrium with numerous setae of which 2+2 shorter medial ones in its apical part. Dens with numerous setae on anterior side and eight setae on posterior side (two basal, three internal and three external) (Fig. 56). Mucro with three teeth (Fig. 53).

Macrosetae differentiated, on last abdominal segments with few cilia (Fig. 45), on Abd.V shorter than length of tergite (ca. 0.6). S-setae thin, well different from common setae, pattern of s-setae complete, s-formula Th.II–Abd.V: 2al+6accp, 1al+6accp / 6 accp, 6 accp, 6accp, 1 am+6 accp, 2 am + 5 accp (Fig. 44). Micro s-setae 1,1/1,1,1 (ms) on Th.II–Abd.III. Micro s-setae of Abd.III shorter than s-setae (Fig. 10). As a rule, two common setae of p-row separate neighbouring accp s-setae. Formula of common setae in p-row between s and ms: 3–4s1–2s2s1s (Abd.I), 3–5s1–2s2s2–3s1–2s1–2s (Abd.II), 3–5s1–3s2–4s2–4s1–2ms0s1–2s (Abd.III), 1–3s2–3s2s1–2s2–4(s)1s (Abd.IV) (Fig. 44). Males present.

#### Affinity.

Due to posterior position of accp4 s-setae on Abd.IV the species, as expected, belongs to Palearctic branch of species of the genus *Parisotoma* (Potapov et al. 2011). Short macrosetae, many s on Ant.1, and increased number of common setae between s-setae in p-row indicate the similarity to *P. appressopilosa* Potapov, 1991, described also from Baikal shore. They differ in number of laterodistal setae on ventral tube (3+3 in *P. baicalica* sp. nov. vs. 2+2 in *P. appressopilosa*), outer setae on lower subcoxa of Leg 1 (one vs. two), shape of claw (more slender in *P. baicalica* sp. nov.), number of s-setae on body tergites (fewer in *P. appressopilosa*) and number of internal setae (three vs. two) on dens. In appearance, the grey *P. baicalica* sp. nov. is well distinguished from the white *P. appressopilosa* if mixed in one site. The new species occurs along Baikal shore where the third congener, *P. reducta* Rusek, 1984, is very common in the forest litter. Both species are grey and can be mixed together in littoral zone. Less distinct eye pigment and shorter macrosetae readily help to discriminate between them. They also differ in size of lateral process of papilla E (Fig. 50 vs. Fig. 51), number of internal setae on dens (Fig. 56 vs. Fig. 57), outer setae on lower subcoxa of Leg 1 and other characters. *P. terricola* Rusek, 1984 (also described from Baikal) and *P. baicalica* sp. nov. share large inner s-setae of AO on Ant.3. Concerning European species, *P. agrelli* (Delamare Deboutteville, 1950) lives on sea shores and most resembles the new species due to short macrosetae, small ommatidia, and 3+3 laterodistal setae on ventral tube. They sharply differ in outer setae on lower subcoxa of Leg 1 (absent in *P. baicalica* sp. nov.) and number of s on Ant.1 (only two in *P. agrelli*).

**Figures 58, 59.**
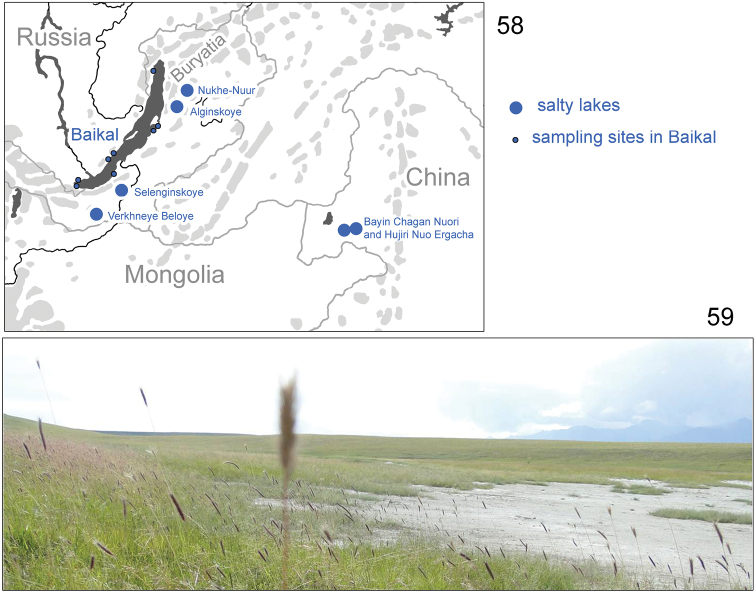
Sampling sites (**58**) and shore of Verkhneye Beloye Lake (**59**).

Slender claw, polychaetosis, short macrosetae, and expanded lateral process of papilla E indicate adaptation to live in contact to fresh water. The combination of 3+3 laterodistal setae on ventral tube and only one outer seta on lower subcoxa of Leg 1 indicate the formal similarity with the eurytopic species *P. notabilis* (rare at Lake Baikal) but *P. baicalica* sp. nov. differs by all “littoral” characters mentioned above.

The value of basal process on labial papilla B (Fig. 52) calls for further study. So far it was not mentioned in the descriptions of labium while we have seen it also in *P. reducta* and *P. appressopilosa* that may suggest its diagnostic importance for the genus *Parisotoma*.

#### Distribution and ecology.

Known only from two distant localities on the Baikal shore. A littoral species.

#### Name derivation.

It is named after the location of the type locality.

##### New species records on the shore of Lake Baikal

*Folsomia uniramia* Potapov & Gulgenova, 2013: Buryat Republic, SE shore of Lake Baikal, at Ust’-Barguzin, 53.4086°N, 108.9879°E, floatation of sand at 5 m distance from water edge, 05.VIII.2014, coll. M. Potapov and A. Gulgenova.

*Scutisotoma baica* Potapov, Babenko & Fjellberg, 2006: Buryat Republic, SE shore of Lake Baikal, at Ust’-Barguzin, 53.4086°N, 108.9879°E, floatation of wet sand at water edge, 05.VIII.2014, coll. M. Potapov and A. Gulgenova.

*Isotomurus stuxbergi* (Tullberg, 1876): Buryat Republic, SE shore of Lake Baikal, 5 km N from Turka, 14.V.2017, coll. A. Gulgenova and S. Gulgenov.

Other new records of species concern shores of saline lakes and therefore are given in the Table of Appendix 1.

##### Faunistic notes

Three ecological groups can be recognised among the recorded species:

1. Species widely distributed in the Holarctic and living also at sites distant from the lake shore (notated as W in Appendix 1). This group mostly consists of xerophilic and steppe species (e.g., F. parvulus, A. stebayevae, A. mongolicus, F. mongolica sp. nov.) which occur also on neighbouring arid landscapes of continental Asia. They often prefer saline lands and penetrate to catenas of saline lakes where they can be numerous. The group also include widely distributed eurytopic (P. notabilis, P. minima) and ruderal species (P. minuta). The latter species can be very abundant in lower part of catenas.
2. Lake species (as L in Appendix 1). This group consists of species found so far only in lake shores. They mostly belong to the fauna of Lake Baikal (P. barathrum sp. nov., S. acorrelata, S. baica, S. robustodens, P. appressopilosa, P. baicalica sp. nov.). Folsomia uniramia presumably belongs to the group since it has been recorded only in dunes at this lake shore. S. acorrelata and P. appressopilosa also occur in shore of saline Alginskoye Lake which is close to Baikal Lake. Considering salt-lake catenas, E. buryatica sp. nov. is only species which belongs to this group.
3. Hygrophilic widely distributed species (as H in Appendix 1). In our materials, Isotomurus stuxbergi (Tullberg, 1876) is the only member belonging to this group. It was found once in the Baikal shore.

## Supplementary Material

XML Treatment for Pseudanurophorus barathrum

XML Treatment for Scutisotoma acorrelata

XML Treatment for Ephemerotoma buryatica

XML Treatment for Folsomia mongolica

XML Treatment for Parisotoma baicalica

## Appendix 1

**Table:** List of species of Isotomidae found at Lake Baikal shore and catenas of saline lakes of Buryatia and Inner Mongolia (Russia and China), based on literature data and new records.

Species*	Lake Baikal	saline lakes of Buryatia				Saline lakes of Inner Mongolia		Source
		1	2	3	4	5	6	
*Anurophorus mongolicus* Dunger, 1982 (W)			+		+			p.p.
*Pseudanurophorus barathrum* sp. nov. (L)	+							p.p.
*Proisotoma minima* (Absolon, 1901) (W)				+				p.p.
*Proisotoma minuta* (Tullberg, 1871) (W)					+		+	p.p.
*Scutisotoma acorrelata* Potapov, Babenko & Fjellberg, 2006 (L)	+	+						Sokolovskaya 1989^P.b.^, Potapov et al. 2006; p.p.
*Scutisotoma baica* Potapov, Babenko & Fjellberg, 2006 (L)	+							Potapov et al. 2006; p.p.
*Scutisotoma fjellbergi* (Dunger, 1982) (W)					+			p.p.
*Scutisotoma robustodens* Huang & Potapov, 2012 (L)	+							Huang and Potapov 2012
*Scutisotoma stepposa* (Martynova, 1975) (W)	+							p.p.
*Ephemerotoma buryatica* sp. nov. (L)					+			p.p.
*Appendisotoma stebayevae* (Grinbergs, 1962) (W)	+				+	+	+	Sokolovskaya 1989, p.p.
*Folsomides parvulus* Stach, 1922 (W)				+		+	+	p.p.
*Folsomides aridoviator* Potapov & Stebaeva, 1997 (W)				+				p.p.
*Folsomia mongolica* sp. nov. (W)						+	+	p.p.
*Folsomia pseudodecemoculata* Stebaeva, 1971 (W)		+		+	+		+	p.p.
*Folsomia paoinflata* Potapov & Stebaeva, 2006 (W)	+							Potapov and Gulgenova 2013
*Folsomia quadrioculata* (Tullberg, 1871) (W)	+			+				Sokolovskaya 1989, p.p.
*Folsomia uniramia* Potapov & Gulgenova, 2013 (L)	+							Potapov and Gulgenova 2013, p.p.
*Isotomurus stuxbergi* (Tullberg, 1876) (H)	+					+	+	p.p.
*Parisotoma appressopilosa* Potapov, 1991 (L)	+	+						Potapov 1991, p.p.
*Parisotoma baicalica* sp. nov. (L)	+							p.p.
*Parisotoma reducta* Rusek, 1984 (W)	+				+			p.p.
*Parisotoma notabilis* (Schäffer, 1896) (W)						+		p.p.

Key: * – species identified only to generic level are not given, p.p. – data of present paper, Lakes: 1 – Alginskoye, 2 – Verkhneye Beloye, 3 – Nukhe-Nuur, 4 – Selenginskoye (= Sulfatnoye), 5 – Bayin Chagan Nuori, 6 – Hujiri Nuo Ergacha, (W) – widely distributed species, (L) – lake species (see the Faunistic notes), (H) – hygrophilic species, P.b. – as *Proisotoma buddenbrocki* and P. cf. buddenbrocki.
